# Disseminated Tuberculosis

**DOI:** 10.4269/ajtmh.24-0132

**Published:** 2024-07-02

**Authors:** Jorge C. F. Nakazaki, Mario Suito

**Affiliations:** ^1^ Instituto de Medicina Tropical ‘Alexander von Humboldt’, Universidad Peruana Cayetano Heredia, Lima, Peru;; ^2^ Facultad de Medicina Alberto Hurtado, Universidad Peruana Cayetano Heredia, Lima, Peru

A 27-year-old male patient with no significant past medical history presented to the emergency department with a 1-year history of intense lower back pain and multiple masses in the inguinal, knee, and hip regions. He had undergone surgical drainage of the inguinal mass with no apparent source of infection. The lower back pain led him to prostration. The patient is originally from Pucallpa, Ucayali (low jungle), but now resides in Lima, Peru. He had no known tuberculosis (TB) contacts. His vital signs were normal. There was a fistula in the right iliac fossa with purulent and bloody drainage. Crackles were heard on the left upper lung. Slightly tender, nonerythematous, cold fluid collections were noted in the left hip and the infrapatellar region of the left and right knees (Figure 1).

**Figure 1.**
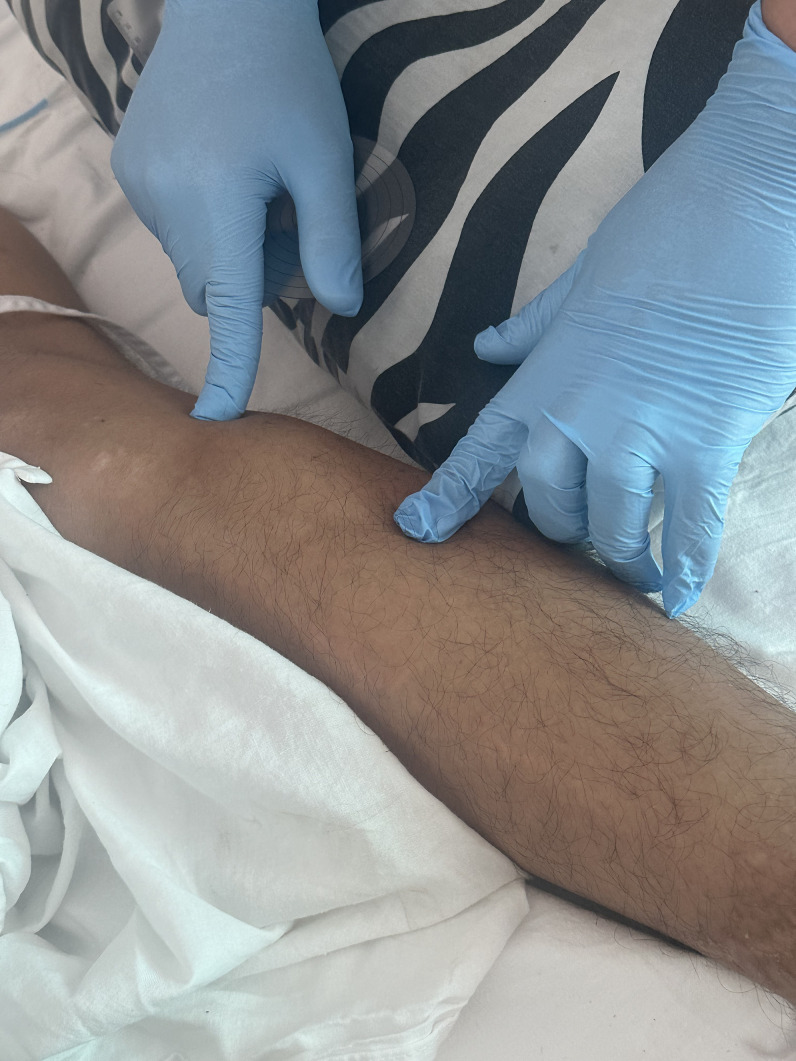
Cold abscess on the left infrapatellar region.

His hemoglobin level was 10.1 mg/dL. Stool ova and parasites were negative, including for *Strongyloides stercoralis*. An HIV test was negative, and a human T-lymphotropic virus 1 (HTLV-1) electrochemiluminescence immunoassay was reactive. X-rays showed periostitis in the left knee, lumbar involvement, and destruction of the left hip joint (Figure 2). Chest X-ray showed cavitation in the left upper lung (Figure 3). Computed tomography revealed a psoas abscess (Figure 4). Samples for culture were collected, and acid-fast bacillus (AFB) staining was done. A Ziehl-Neelsen stain of fluid aspirated from the left infrapatellar collection was 1+ positive (Figure 5). An auramine stain of fluid aspirated from the left psoas abscess was positive, and a GeneXpert MTB/RIF ULTRA assay detected *Mycobacterium tuberculosis* (MTB) with no rifampicin resistance. His body mass index was 25.6 kg/m^2^.

**Figure 2.**
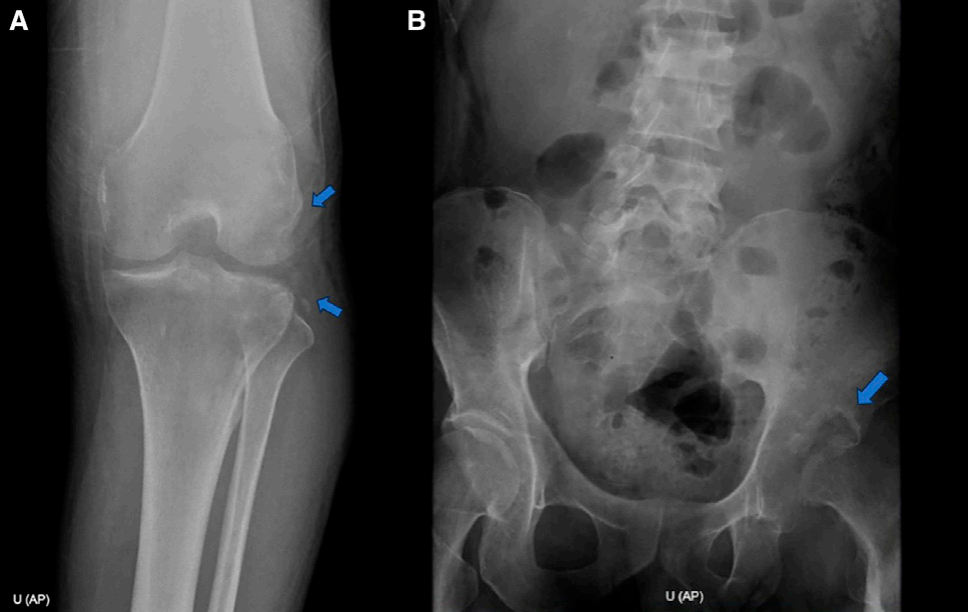
(**A**) Periostitis of the left knee (arrows). (**B**) Destruction of the left hip joint (arrow).

**Figure 3.**
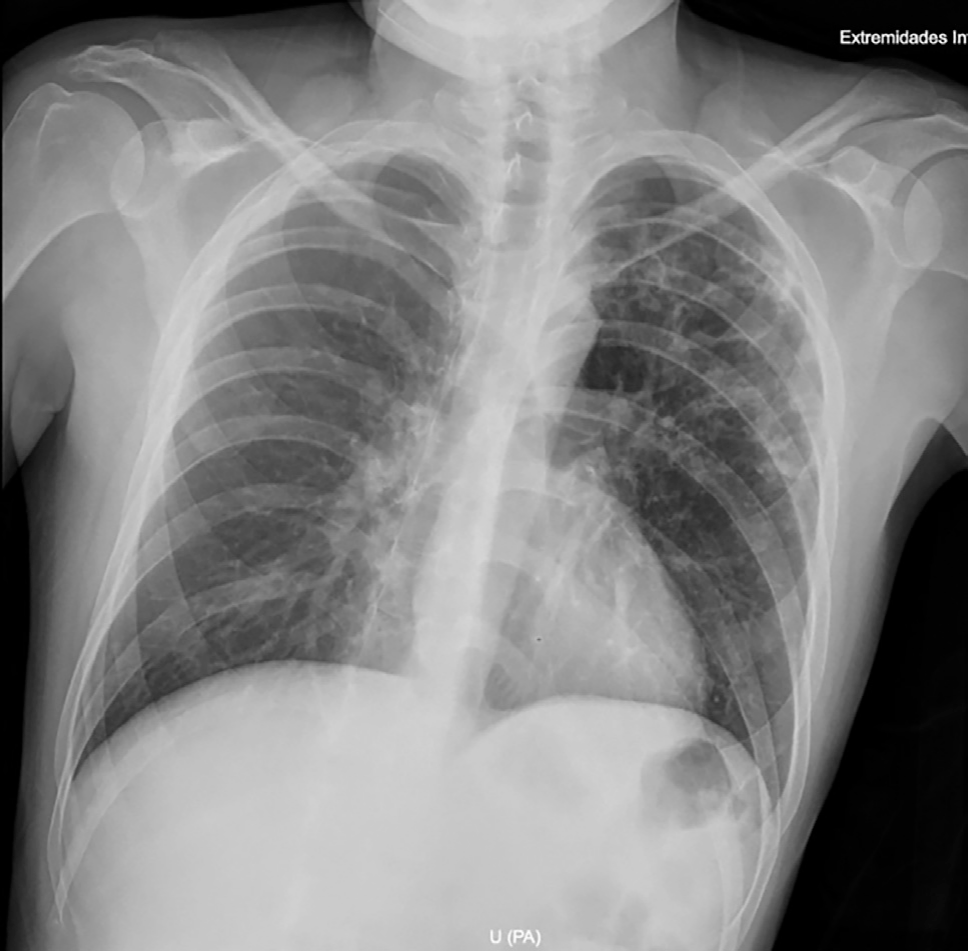
Chest X-ray showed cavitation in the upper left region and nearby smaller ones.

**Figure 4.**
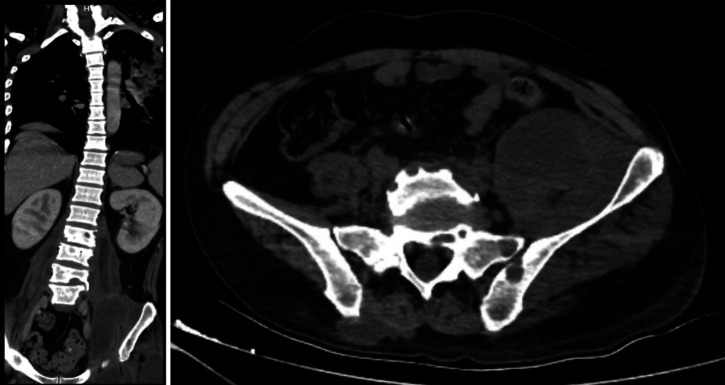
Left psoas abscess in two different views.

**Figure 5.**
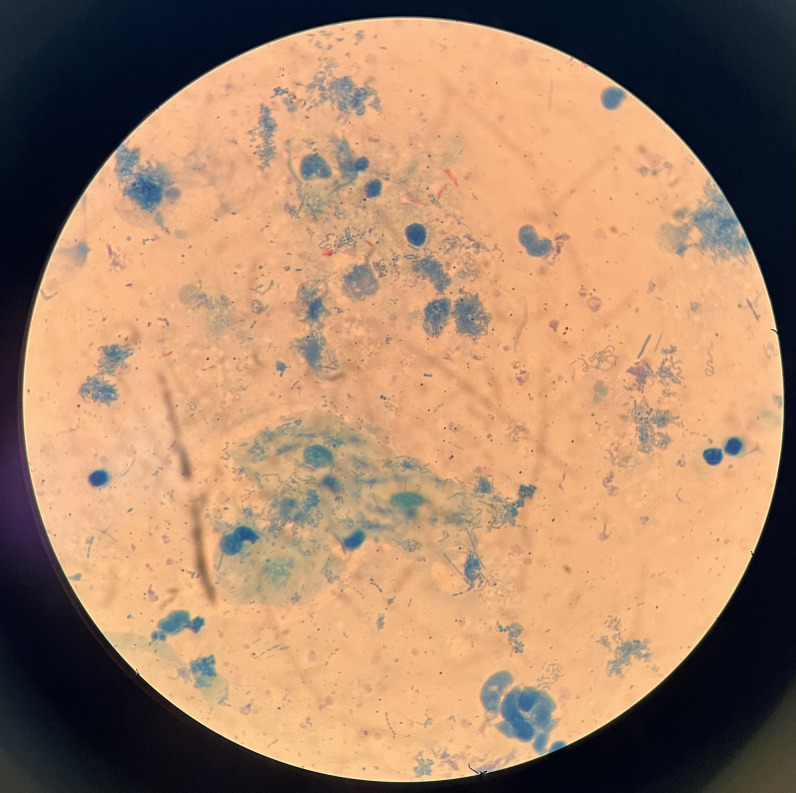
Acid-fast bacillus staining of the left cold abscess was positive.

Immunosuppressed patients are at higher risk of developing disseminated TB.^1^ A study from Lima described an increased prevalence of TB-related family deaths and an increased prevalence of 3+ AFB sputum smears among HTLV-1–infected patients.^2^ Human T-lymphotropic virus 1 alters cells by affecting transcription factors and signaling cascades. The Tax protein impacts NFAT and regulates interleukin-2 transcription, affecting T cells’ normal functioning. Extrapulmonary TB can affect mainly lymph nodes and pleural and skeletal areas. Half of skeletal TB cases are Pott’s disease, which can cause vertebral osteomyelitis and complications such as secondary psoas abscess. Delayed treatment can lead to femoral head necrosis or fistula formation.^3^ Extrapulmonary TB is treated similarly to pulmonary TB, but because of bone involvement, the treatment is longer. Debridement or drainage may be necessary for cold abscesses. Early diagnosis and treatment can lead to a favorable prognosis.^4^^,^^5^

## References

[1] Kwan C Ernst JD, 2011. HIV and tuberculosis: A deadly human syndemic. Clin Microbiol Rev 24: 351–376. 21482729 10.1128/CMR.00042-10 PMC3122491

[2] Verdonck K González E Henostroza G Nabeta P Llanos F Cornejo H Vanham G Seas C Gotuzzo E, 2007. HTLV-1 infection is frequent among out-patients with pulmonary tuberculosis in northern Lima, Peru. Int J Tuberc Lung Dis 11: 1066–1072. 17945062

[3] Malhotra MK, 2012. Cold abscess of the anterior abdominal wall: An unusual primary presentation. Niger J Surg 18: 22–23. 24027388 10.4103/1117-6806.95481 PMC3716239

[4] Dartois VA Rubin EJ, 2022. Anti-tuberculosis treatment strategies and drug development: Challenges and priorities. Nat Rev Microbiol 20: 685–701. 35478222 10.1038/s41579-022-00731-y PMC9045034

[5] Lee JY, 2015. Diagnosis and treatment of extrapulmonary tuberculosis. Tuberc Respir Dis (Seoul) 78: 47–55. 25861336 10.4046/trd.2015.78.2.47 PMC4388900

